# Association of *N*-nitrosodimethylamine exposure with cognitive impairment based on the clues of mice and humans

**DOI:** 10.3389/fnagi.2023.1137164

**Published:** 2023-06-27

**Authors:** Wei Liu, Jia Huang, Zhi Yan, Yankui Lin, Guanqin Huang, Xiao Chen, Zhou Wang, Peter S. Spencer, Jianjun Liu

**Affiliations:** ^1^Shenzhen Key Laboratory of Modern Toxicology, Shenzhen Medical Key Discipline of Health Toxicology (2020–2024), Shenzhen Center for Disease Control and Prevention, Shenzhen, China; ^2^Key Laboratory of Molecular Epidemiology of Hunan Province, School of Medicine, Hunan Normal University, Changsha, China; ^3^Department of Communicable Diseases Control and Prevention, Shenzhen Center for Disease Control and Prevention, Shenzhen, China; ^4^Food Inspection and Quarantine Center, Shenzhen Customs, Shenzhen, China; ^5^Department of Nutrition and Food Safety, Shenzhen Center for Disease Control and Prevention, Shenzhen, China; ^6^Department of Neurology, School of Medicine, Oregon Institute of Occupational Health Sciences, Oregon Health and Science University, Portland, OR, United States

**Keywords:** *N*-nitrosodimethylamine, cognitive impairment, risk assessment, formaldehyde, mouse, elderly

## Abstract

*N*-nitrosodimethylamine (NDMA) is an environmental and food contaminant, but limited data to concern whether NDMA has adverse effects on the brain. This study first determined the concentration of NDMA in foods from aquaculture markets in Shenzhen, then analyzed the effects on C57BL/6 mice and further evaluated on the urine samples of elderly Chinese residents with normal cognition (NC, *n* = 144), cognitive decline (CD, *n* = 116) and mild cognitive impairment (MCI, *n* = 123). The excessive rate of NDMA in foods was 3.32% (27/813), with a exceeding range of 4.78–131.00 μg/kg. Behavioral tests showed that 60 days treatment of mice with 3 mg/kg NDMA reduced cognitive performance. Cognitive impairment in human was significantly associated with sex, educational levels, length of residence in Shenzhen, household registration, passive smoking, rice, fresh vegetables, bacon products. NDMA was detected in 55.4% (212/383) of urine samples, with a median concentration of 0.23 μg/L (1.20 × 10 ^–7^–157.39 μg/L). The median concentration for NC, CD and MCI were 0.32, 0.27, and 0 μg/L, respectively. The urinary NDMA concentration had a strong negative correlation with cognitive impairment (*Kendall’s Tau-b* = −0.89, *P* = 0.024). The median estimated daily intake (EDI) of NDMA was determined to be 6.63 ng/kg-bw/day. Taken together, there appears to be an association between NDMA and human and murine cognition, which provides a new clue to Alzheimer’s disease (AD).

## Introduction

Alzheimer’s disease (AD), with memory loss as the main clinical feature, is the most common type of senile dementia and seriously threatens the physical and mental health of elderly people worldwide (Hodson, 2018). AD is preceded by mild cognitive impairment (MCI), which manifests as age-inappropriate memory impairment and/or other cognitive impairment (Sanford, 2017). MCI has become an increasingly serious issue for families and society, such that interest in AD prevention has gradually shifted to the recognition of early MCI (Norton et al., 2014; Angevaare et al., 2022). If the environmental contributions to this progressive brain disorder can be discovered, primary disease prevention may become a realistic goal.

Nitrosamines have long been recognized as a class of highly carcinogenic compounds, the simplest of which is the probable human carcinogen *N*-nitrosodimethylamine (NDMA) (U.S. Environmental Protection Agency, 2002, 2014). Human exposure to NDMA is widespread across the globe. NDMA is generated endogenously from precursors (such as amines and nitrites) in food and drinking water. External sources of NDMA include food items, water, cigarette smoke, and to a lesser extent rubber products, toiletry and cosmetic products, pesticides and contaminated medications (U.S. Agency for Toxic Substances and Disease Registry [ATSDR], 2022). NDMA and other *N*-nitrosamine contaminants have also been recently reported in multiple pharmaceuticals (Adamson and Chabner, 2020; Zmysłowski et al., 2020). A contemporary study of adult residents in 20 Chinese provinces revealed a range of NDMA intake (171 to 425 ng/d) from food and drinking water, the latter contributing about 13% of the total (Li X. et al., 2021). The average total NDMA intake per capita in China (251 ng/d) was higher than that in the United States (136 ng/d) and Canada (87.6 ng/d), with higher NDMA intake in relatively affluent coastal provinces (notably from aquatic products) compared to inland provinces (Li X. et al., 2021). The United States (U.S.) Agency for Toxic Substance and Disease Registry (ATSDR) considers there are insufficient data to establish a chronic human (cancer) Minimum Risk Level (MRL), but the proposed acute MRL level for oral exposure to NDMA is 0.00001 mg/kg/day (U.S. Agency for Toxic Substances and Disease Registry [ATSDR], 2022).

While the carcinogenic properties of nitrosamines are widely recognized, a role for the compound in the etiology of neurodegenerative diseases is largely untested. The present study explores the previously untested hypothesis that exposure to nitrosamines (R^1^N(-R^2^)-*N* = O) may play a critical role in the pathogenesis of sporadic AD (de la Monte and Tong, 2009) and related neurodegenerative disorders of environmental origin (Spencer, 2019). Epidemiological trends indicate that increasing AD mortality in the U.S. mainland correlate with trends in the consumption of processed foods, use of preservatives and reliance on nitrogen-containing fertilizers that contain nitrosamines and related genotoxins (de la Monte et al., 2009). *N*-Nitrosodiethylamine (NDEA) as a typical N-nitroso compound, and is responsible for the changes in the nuclear enzymes associated with DNA repair/replication. NDEA-treated laboratory rats developed cognitive impairment, AD-type neurodegeneration, and brain insulin resistance (Tong et al., 2009). NDEA (15–250 μg/mL) produced similar effects in post-mitotic cerebellar granule cell cultures, notably concentration-dependent impairments in ATP production and mitochondrial function, and increased levels of oxidative DNA damage, p-Tau, and amyloid-beta protein precursor-amyloid-beta (APP-Aβ) (de la Monte and Tong, 2009). Methylazoxymethanol (MAM), a structural analog of *N*-nitroso compounds, is also a potent alkylating agent and probable key trigger of Western Pacific amyotrophic lateral sclerosis and Parkinsonism-dementia complex (ALS/PDC) (Kisby et al., 2011; Spencer et al., 2020). However, few studies have reported the possible relationship between NDMA exposure levels and neurodegenerative diseases in human samples. Taken in concert, these studies indicate the need to determine whether nitroso compounds are potential risk factors for human neurodegenerative diseases.

So the present study was carried out in the coastal Chinese city of Shenzhen with three objectives: (1) to measure the NDMA content of commercially available foods from aquaculture markets and assess food-borne exposure levels, (2) to examine the effects of sub-chronic oral NDMA treatment on mice cognitive function in controlled experimental studies, and (3) to determine NDMA in human urine as an estimate of internal exposure and evaluate the association of exposure level with cognitive function of residents. It is expected to provide a new strategies for AD.

## Materials and methods

### Chemicals

Samples of NDMA (99% purity) were obtained from Sigma-Aldrich (Shanghai) Trading Co., Ltd., (Shanghai, China). Listed NDMA impurities included: methanol. Samples of methylazoxymethanol acetate (MAM, 98% purity) were obtained from FUJIFILM Wako Pure Chemical Corporation (Chuo-Ku, Osaka, Japan). Listed MAM impurities included: methanol. Other organic solvents like methanol and acetonitrile, which were analytical grade, were provided by Merck (Darmstadt, Germany). Water was prepared by purification systems (Millipore Co., Ltd., Billerica, MA, USA).

### Monitoring of NDMA in foods

A total of 813 food samples was randomly selected in Shenzhen aquaculture markets during the period of 2017 to 2021. Samples included meatballs, sausages, bacon, squid, cuttlefish, sandworms, shrimp, and pollock filets, among other food items. NDMA content was determined by gas chromatographic mass spectrometry (TSQ Quantum GC-MS, Thermo Fisher, Waltham, MA, USA) according to the National Standard for Food Safety (GB 5009.26-2016) Measurement of *N*-nitrosamines in food. Briefly, NDMA in 200 g samples was extracted by steam distillation and dichloromethane and 250 mL of the extraction liquid was then concentrated to 1 mL prior to analysis.

### Animal study

Animal treatment were conducted in accordance with the ethics principles of laboratory animal care and use (National Research Council (US) Committee for the Update of the Guide for the Care and Use of Laboratory Animals, 2011). The study design was approved by the Medical Ethics Committee of Shenzhen Center for Disease Control and Prevention (No. 2020015). Efforts were made to minimize animal suffering and the number of animals used for experiments. Food and water were provided *ad libitum*.

Newly lactated C57BL/6 mice (age 21–22 days, weight 11–12 g) were purchased from Guangdong Medical Laboratory Animal Center (Quality certificate number 44007200078699). After 1 week of quarantine observation, the mice were divided into five groups: normal saline control group, 0.03 mg/kg NDMA low-dose group, 0.3 mg/kg NDMA medium-dose group, 3 mg/kg NDMA high-dose group, and 2 mg/kg MAM positive control group. The NDMA dose was based on the National Standard for food safety (GB2762-2017) Limit of contaminants in food. 30 mice in each group, half male and half female. Mice were treated with the test substances by intraperitoneal injection (*i.p.*) every other day for 2 months.

### Behavioral test

After 60 days of treatment, the spatial learning and memory of mice was assessed using the Morris water maze (MWM) test (Vorhees and Williams, 2006). The MWM test included the place navigation trail and the spatial probe trail. The place navigation trail lasted for 5 days. Each mouse was trained 4 times a day for 60 s per session (training period). On Day 7, the spatial probe trail was used to measure memory ability (test period). Specifically, the MWM test consists of a round pool with a diameter of 150 cm and a height of 60 cm. The inside of the pool is painted black with different shapes of markers. The water temperature in the pool is controllable, the light in the room is constant, and there is no direct light in the pool. The pool is divided into four quadrants with four equidistant points on the pool wall. In the target quadrant, a circular black platform with a diameter of 12 cm and a height of 23 cm is placed 30 cm away from the pool wall. The platform is located 1–2 cm below the water surface. A camera connected to the display system is installed above the maze to record the movement track of mice synchronously. Mice are required to use distal cues to navigate from start locations around the perimeter of an open swimming arena to locate a submerged escape platform. An animal behavior video analysis system (Xeye V3.20, Beijing Macroambition S&T Development Co., Ltd., Beijing, China) was used to identify and analyze the behavior of mice, including: the latent period, swimming track chart, average speed, crossing times, time of target quadrant, and distance of target quadrant, among other measures.

### Brain histopathology examination

After the behavioral test, mice were anesthetized with 10% chloral hydrate and systemically perfused via the left ventricle with normal saline to remove circulating blood. The whole brain was removed, fixed in 4% paraformaldehyde for 48 h, dehydrated stepwise with ethanol, immersed in xylene for 30 min and then embedded in paraffin. Continuous sagittal brain sections (each 5 μm) were stained with hematoxylin and eosin (HE) and imaged by laser scanning confocal microscopy (Leica, Wetzlar, Germany). The slides were examined by investigators blind to the treatment groups.

### Subjects

**FIGURE 1:**
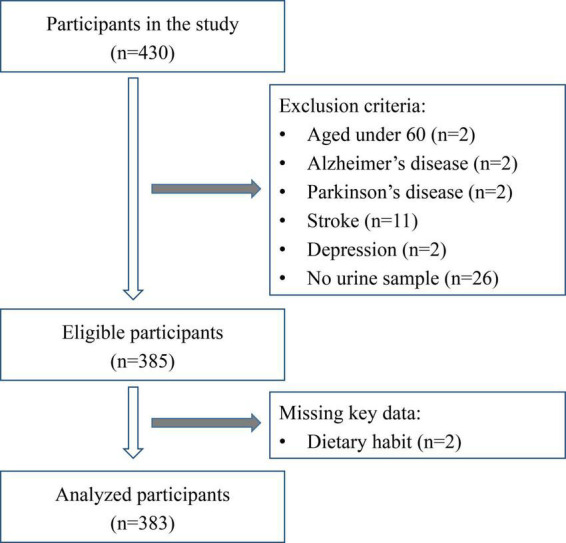
Flowchart of inclusion and exclusion process of participants in the study.

Study approval was granted by the Ethics Committee of Shenzhen Center for Disease Control and Prevention (No. 2019027B). Between February and May 2019, elderly people were recruited randomly in Luohu District, Shenzhen, China. Subjects were selected for study according to the following criteria. Inclusion criteria included: (I) 60 years of age or older; (II) living in Shenzhen community for 1 year or more; (III) good eyesight, hearing and comprehension, able to complete face-to-face interview; (IV) informed consent, good compliance; (V) able to complete questionnaire data, physical examination and provide a urine sample. Exclusion criteria included: (I) suffering from mental illness, sensory disability (deafness, blindness), stroke, dementia or severe hearing and visual impairment, among other disabilities; (II) poor compliance, did not sign informed consent; (III) incomplete questionnaire and physical examination data. The inclusion and exclusion process is shown in Figure 1. A total of 383 subjects was included in the study.

### Questionnaire and physical examination

The questionnaire, which was administered to each participant by face-to-face interview, sought information on sociodemographic characteristics (gender, age, education level, length of residence, and household registration), disease history (hypertension, hyperlipidemia, diabetes mellitus, chronic hepatitis, and cancer), medication history and lifestyle (cigarette, alcohol drinking, and dietary characteristics).

The physical examination assessed blood pressure, serology, and medication use. Hypertension was defined as diastolic blood pressure ≥90 mmHg and/or systolic blood pressure ≥140 mmHg, self-reported hypertension diagnosed by a physician, or use of drugs indicated for hypertension. Hyperlipidemia was defined as total cholesterol ≥6.2 mmol/L, triglyceride level ≥2.3 mmol/L and/or self-report of hyperlipidemia diagnosed by a physician or reported taking lipid-lowering drugs. Diabetes mellitus was defined as a fasting blood glucose value ≥7.0 mmol/L, use of antidiabetic drugs or self-report of a clinical diagnosis of diabetes. Chronic hepatitis and cancer were defined as use of disease related drugs for more than 6 months or self-reported clinical diagnosis by a physician.

### Definition and grouping of cognitive function assessment

The cognitive function of subjects was assessed by Mini-Cog screening and the Mini-Mental State Examination (MMSE) according to the standards of cognitive assessment (Chinese Guidance group for diagnosis and treatment of dementia and Cognitive Impairment, 2018; Li et al., 2018). The Mini-Cog full score is five, which is considered to reflect normal cognitive function, while a subject scoring <5 is considered to have cognitive dysfunction, such that further MMSE examination is required. Thirty is the highest MMSE score, which is related to education level. Those with a MMSE score >20 for primary school and below or >26 for middle school education and above were considered to have cognitive decline (CD), or pre-MCI. MMSE scores of ≤20 for those with up to and including primary school education or ≤26 for those with middle school education and beyond were defined as having MCI. Using this classification, subjects were divided into three groups for analysis: normal cognition (NC) group, CD group, and MCI group.

### Urinary NDMA determination

Approximately 4 mL of mid-stream morning urine was collected from each participant and preserved at −20 C. Urinary concentrations of NDMA were measured by high-performance gas chromatography tandem mass spectrometer (TSQ Quantum GC-MS/MS, Thermo Fisher, Waltham, MA, USA), according to published methods (Guo et al., 2013; Seyler et al., 2013) with minor modification. Briefly, an equal amount of ultra-pure water was first added to the urine sample, followed by 25 μL of 1 mg/L NDMA-D6 internal standard. The NDMA external standard was 100 μg/L. After adding 10 mL acetonitrile, the samples were shaken at 2000 r/min for 20 min and then centrifuged at 9500 r/min for 10 min. Eight mL of supernatant was placed into a 15 mL centrifuge tube and blown down to 1 mL with nitrogen. The fluid was finally filtered through a 0.22 μm organic membrane and positioned on the instrument for NDMA detection.

Instrumental analysis employed a Stabilwax chromatographic column (30 m × 0.25 mm × 0.25 μm, Thermo Fisher, Waltham, MA, USA) and multiple reaction monitoring (MRM). The inlet temperature was 230 C. Injection mode: non-split injection (1 min). Column temperature procedure: 60 C for 2 min, then 8 C/min to 140 C for 8 min, and then 40 C/min to 240 C for 10 min. Electron ionization mode. Ion source temperature of 200 C; solvent delay time of 5.5 min and injection volume of 2 μL.

### Quality assurance and quality control (QA/QC)

Questionnaires were checked for completion, errors were corrected, and data were entered twice into EpiData 3.1 by two separate investigators. For sample analysis, the blanks were measured every batch of 20 samples and determined to be below the limit of NDMA quantification (LOQ). The internal standard with moderate concentrations was applied to confirm the stability of instrument responses. The standard deviation was observed to be <10%. The calibration curves in the range of 0.5–100 μg/L for all target compounds showed linearity with regression coefficients (R^2^) > 0.99.

### Estimated daily intake of NDMA and health risks

The human internal exposure level of NDMA as a potential human liver carcinogen was estimated based on the detected concentration in urine (World Health Organization, and Food and Agriculture Organization of the United Nations, 2009; Fernández et al., 2020; Li K. et al., 2021). Estimated daily intake (EDI) of NDMA in food was calculated according to the following formula: EDI = *Cu* × *Vu* × 1000/BW, where *Cu* (μg/L) denotes urinary concentration of NDMA, *Vu* (L/day) for average daily urine volume (i.e., 1.7 L/day) and BW (kg) the average body weight (i.e., 60 kg) for Chinese elderly, respectively.

A hazard quotient (HQ) was used to assess the risk of cognitive impairment from NDMA exposure according to the formula (Sang et al., 2019; Li K. et al., 2021): HQ = EDI/RfD. An HQ > 1 indicates that the estimated exposure level exceeds the referenced exposure level, thus indicating a potential human health risk. The EDI (mg/kg-bw/day) corresponds to the estimated daily intake of NDMA. The RfD (mg/kg-bw/day) or Reference Dose, is defined as an estimate of the daily exposure that is likely to be without appreciable risk of deleterious effect during a lifetime. U.S. Environmental Protection Agency (EPA) appears not to have published a chronic oral reference dose (RfD) or a chronic inhalation reference concentration (RfC) for NDMA. EPA lists a risk-associated dose (RAD) (U.S. Environmental Protection Agency, 1999) of 2.0 × 10^–7^ mg/kg/day that was used in replace a RfD in the present study.

### Statistical analysis

Quantification of individual analytes was calibrated by plotting the ratio of the analyte signal to the internal standard signal as a function of the concentration of the analyte standards. For concentrations less than the LOQ, one half of the LOQ was applied to substitute in analyses with statistical software SPSS 22.0. The counting data were expressed as a percentage. The reference values for urinary NDMA were set at the medians. Chi-square test was used for the comparisons of categorical variables. One-Way Analysis of Variance (ANOVA) was used for the comparisons of continuous variables. The Brown-Mood Median test served to compare median variables. The Jonckheere-Terpstra test for ordered differences was used to compare trends of multiple groups. Kendall’s Tau-b correlation analysis was used to determine whether there was a correlation between cognitive impairment and urinary NDMA concentration. Using cognitive impairment as the dependent variable, the factors with statistical significance in intergroup comparison were screened stepwise and analyzed by a multivariate non-conditional Logistic regression model. Two-sided *P*-values < 0.05 were considered statistically significant.

## Results

### NDMA concentration in foods

The content of NDMA in foods was determined by gas chromatography-mass spectrometry (GC-MS) according to a national standard (GB 5009.26-2016). NDMA was detected in 79 samples collected between 2017 and 2021. The detection rate was 9.72% (79/813). According to the National Standard Limit Value for contaminants in food (GB2762-2017), 27 samples exceeded the standard limit value of NDMA. The exceedance rate was 3.32% (27/813), with a exceeding range of 4.78–131.00 μg/kg. The exceeding samples mainly involved Chinese sausage and seafood items (squid, cuttlefish, and shrimp) (Table 1).

**TABLE 1:** 2017–2021 *N*-nitrosodimethylamine (NDMA) monitoring status of food items in Shenzhen.

Year	Food number	Excess number	Exceedance range (μg/kg)	Exceedance rate (%)	Main types of exceeding food
2017	135	3	15.80∼35.80	2.22	Shrimp, otcopus, jumbo squid
2018	269	18	5.02∼131.00	6.69	Shrimp, squid, cuttlefish
2019	53	2	4.78∼16.40	3.77	Sausage, shredded squid
2020	215	2	18.40∼20.40	0.93	Shredded squid, pollock filets
2021	141	2	5.17∼8.49	1.42	Squid, fish cake
Total	813	27	4.78∼131.00	3.32	Squid

According to National Standards for Food Safety, limits for contaminants in food (GB2762-2017), the limit of NDMA in meat and meat products is 3.0 μg/kg, and the limit of NDMA in aquatic animals and their products is 4.0 μg/kg.

### NDMA effects on cognitive function of C57BL/6 mice

**FIGURE 2:**
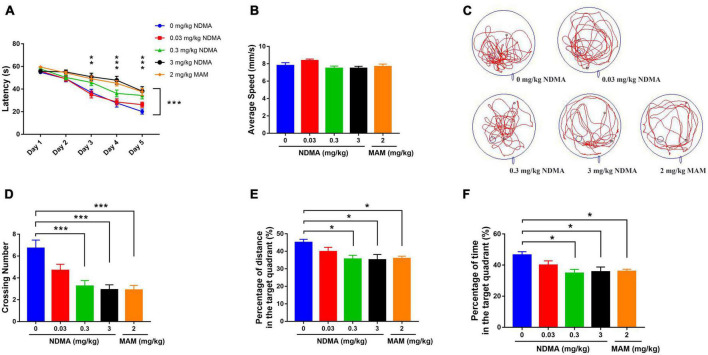
MWM test results to access the cognitive function of mice treated with NDMA (0–3 mg/kg) or MAM (2 mg/kg) in saline (*n* = 23–29 per group). **(A)** Escape latency of the place navigation trail. With the increase of training time, the difference of spatial learning ability of mice in each group was statistically significant (**p < 0.01, ***p < 0.001, analyzed by two-way repeated measures ANOVA). **(B)** Average speed of the spatial probe trail. No differences were observed among groups. **(C)** Representative trajectory diagrams; **(D)** number of platform crossings in the spatial probe trail. Compared with the blank control group, the crossing number in medium and high-dose NDMA group and MAM group was significantly reduced (***p < 0.001, analyzed by one-way ANOVA). **(E)** Percentage of the distance in the target quadrant. Compared with the blank control group, the percentage of travel distance in the target quadrant of mice in the medium and high-dose NDMA group and the MAM group were significantly shortened (*p < 0.05, analyzed by one-way ANOVA). **(F)** Percentage of the time spent in the target quadrant. Compared with the blank control group, the percentage of time in the target quadrant in the medium and high-dose NDMA group and the MAM group were also significantly shortened (*p < 0.05, analyzed by one-way ANOVA). MWM, Morris Water Maze; MAM, methylazoxymethanol. Error bar, SEM.

In view of the high risk of NDMA exposure, we first observed its effect on cognitive function at the animal level. We assessed the cognitive effects after intraperitoneal treatment of C57BL/6 mice with NDMA vs. saline every other day for 60 days, both of which were well tolerated (Figure 2). During the place navigation trail (5 days of training period) of the Morris water maze (MWM) test, statistically significant differences in spatial learning ability were observed for the different concentrations of NDMA and the MAM positive control group (Figure 2A, *F* = 10.31, *p* < 0.001). The high-dose NDMA group and the MAM group were the worst performers. With the increase of training time, the difference of spatial learning of mice in each group was also statistically significant (*F* = 123.6, *p* < 0.001). Compared with the blank control group and the low-dose NDMA group, the high-dose NDMA group and the MAM group were also significantly impaired. In addition, there was an interaction between the treatment groups and time (*F* = 3.127, *p* < 0.001).

During the spatial probe trail of the MWM test, in which the platform is removed, no significant difference was observed in exercise ability among groups (Figure 2B). However, significant differences were observed in the movement trajectories of each group (Figure 2C). Compared with the vehicle control group, the number of crossing platform of mice in the medium- and high-dose NDMA treatment group and the MAM group was significantly reduced (Figure 2D, *F* = 8.106, *p* < 0.001). Moreover, the percentage of travel distance and retention time in the target quadrant (original platform area) in these three groups of mice were also significantly shortened relative to the vehicle control group (Figures 2E, F).

**FIGURE 3:**
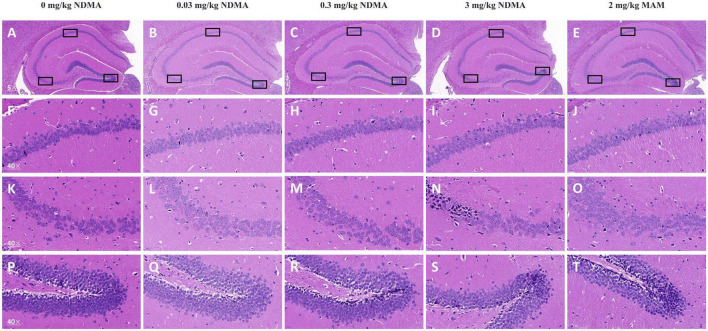
Vertically, there are five columns, and the three graphs below each column are enlarged versions of the three marked black box positions in the first graph above. Boxes outline CA1 (upper left), CA2/CA3 (left), and dentate gyrus (right). **(A–E)** Representative images of HE-stained hippocampus sections shown at 50× magnification. Scale bars = 200 μm. *n* = 4 per group. **(F–J)** Representative images of HE-stained CA1 sections shown at 400× magnification. Scale bars = 20 μm. **(K–O)** Representative images of HE-stained CA2/CA3 sections shown at 400× magnification. Scale bars = 20 μm. **(P–T)** Representative images of HE-stained DG sections shown at 400× magnification. Scale bars = 20 μm.

The results of histopathological examination of the hippocampus were shown in Figure 3. Compared with the vehicle control group, no obvious or little neuronal or other abnormalities of cell morphology were found in mice treated with high-dose NDMA and MAM.

### Characteristics of participants

A total of 383 subjects was enrolled in the study (Figure 1), including 144 in the NC group, 116 in the CD group and 123 in the MCI group (Table 2). Significant differences were found among these three groups with respect to gender, educational levels, length of residence and household registration in Shenzhen (all *P* < 0.05). No significant differences were observed among the groups with respect to age, disease history (including hypertension, hyperlipidemia, diabetes mellitus, chronic hepatitis, and cancer) or medication history (all *P* > 0.05).

**TABLE 2:** Characteristics of the participants by cognitive function status in the study.

Variables	Total (*n* = 383)	Status of cognitive function			*P*-values
		**NC (*n* = 144)**	**CD (*n* = 116)**	**MCI (*n* = 123)**	
Gender (*n*, %)					0.004^a^
Male	157 (41.0)	73 (50.7)	46 (39.7)	38 (30.9)	
Female	226 (59.0)	71 (49.3)	70 (60.3)	85 (69.1)	
Age (years, mean ± SD)	70.27 ± 6.00	70.40 ± 5.70	69.55 ± 5.95	70.81 ± 6.36	0.255^b^
Educational levels (*n*, %)					0.000^a^
Primary school or illiteracy	109 (28.4)	29 (20.1)	49 (42.2)	31 (25.2)	
Middle school	127 (33.2)	42 (29.2)	29 (25.0)	56 (45.5)	
High school or higher	147 (38.4)	73 (50.7)	38 (32.8)	36 (29.3)	
Length of residence (years, *n*, %)					0.026^a^
1–9	92 (24.0)	28 (19.4)	31 (26.7)	33 (26.8)	
10–19	108 (28.2)	42 (29.2)	33 (28.4)	33 (26.8)	
20–29	65 (17.0)	18 (12.5)	17 (14.7)	30 (24.4)	
30–78	118 (30.8)	56 (38.9)	35 (30.2)	27 (22.0)	
Household registration (*n*, %)					0.029^a^
No	147 (38.4)	45 (31.2)	44 (37.9)	58 (47.2)	
Yes	236 (61.6)	99 (68.8)	72 (62.1)	65 (52.8)	
Hypertension (*n*, %)^†^					0.566^a^
No	226 (59.0)	80 (55.6)	71 (61.2)	75 (61.0)	
Yes	157 (41.0)	64 (44.4)	45 (38.8)	48 (39.0)	
Hyperlipidemia (*n*, %)^†^					0.245^a^
No	336 (87.7)	130 (90.3)	103 (88.8)	103 (83.7)	
Yes	47 (12.3)	14 (9.7)	13 (11.2)	20 (16.3)	
Diabetes mellitus (*n*, %)^†^					0.256^a^
No	308 (80.4)	114 (79.2)	99 (85.3)	95 (77.2)	
Yes	75 (19.6)	30 (20.8)	17 (14.7)	28 (22.8)	
Chronic hepatitis (*n*, %)^†^					0.482^c^
No	381 (99.5)	143 (99.3)	116 (100)	122 (99.2)	
Yes	2 (0.5)	1 (0.7)	0 (0)	1 (0.8)	
Cancer (*n*, %)^†^					0.467^c^
No	370 (96.6)	140 (97.2)	110 (94.8)	120 (97.6)	
Yes	13 (3.4)	4 (2.8)	6 (5.2)	3 (2.4)	
Medication history (*n*, %)					0.555^a^
No	334 (87.2)	128 (88.9)	98 (84.5)	108 (87.8)	
Yes	49 (12.8)	16 (11.1)	18 (15.5)	15 (12.2)	

NC, normal cognition; CD, cognitive decline; MCI, mild cognitive impairment; SD, standard deviation.

^a^Chi-square test was used for the comparisons of categorical variables.

^b^One-Way ANOVA was used for the comparisons of continuous variables.

^c^Fisher’s exact test was used.

^†^The disease was defined as self-reported disease.

In terms of lifestyle and dietary habits (Table 3), significant differences were found among the three groups with respect to passive smoking and food use of rice, fresh vegetables and bacon products (all *P* < 0.05). No significant differences were observed among the groups in regard to smoking status, alcohol drinking status and other consumption of food items including: wheat, coarse food, fruits, meat, fish, shrimp/crabs, shellfish, cephalopods, eggs, dairy products, bean products, and pickled vegetables (all *P* > 0.05).

**TABLE 3:** Lifestyle and dietary habits of the participants by cognitive function status in the study.

Variables	Total (*n* = 383)	Status of cognitive function			*P*-values
		**NC (*n* = 144)**	**CD (*n* = 116)**	**MCI (*n* = 123)**	
Smoking status (*n*, %)					0.077^a^
Non-smokers	298 (77.8)	104 (72.2)	91 (78.4)	103 (83.7)	
Smokers	85 (22.2)	40 (27.8)	25 (21.6)	20 (16.3)	
Passive smoking (*n*, %)					0.046^a^
No	326 (85.1)	130 (90.3)	92 (79.3)	104 (84.6)	
Yes	57 (14.9)	14 (9.7)	24 (20.7)	19 (15.4)	
Alcohol drinking status (*n*, %)					0.136^a^
Non-drinkers	321 (83.8)	122 (84.7)	91 (78.4)	108 (87.8)	
Drinkers	62 (16.2)	22 (15.3)	25 (21.6)	15 (12.2)	
Rice (times/month, mean ± SD)	46.14 ± 20.55	44.53 ± 19.79	50.75 ± 20.56	43.68 ± 20.86	0.014^b^
Wheat (times/month, mean ± SD)	20.01 ± 16.21	19.46 ± 15.17	19.79 ± 15.04	20.87 ± 18.40	0.767^b^
Coarse food (times/month, mean ± SD)	17.30 ± 15.40	15.99 ± 15.10	17.21 ± 14.90	18.90 ± 16.16	0.306^b^
Fresh vegetables (times/month, mean ± SD)	40.91 ± 18.89	37.11 ± 18.52	43.18 ± 19.78	43.20 ± 17.86	0.009^b^
Fruits (times/month, mean ± SD)	23.77 ± 14.71	23.74 ± 14.77	24.06 ± 14.30	23.53 ± 15.13	0.962^b^
Meat (times/month, mean ± SD)	28.38 ± 19.29	27.08 ± 18.74	30.91 ± 19.51	27.51 ± 19.62	0.236^b^
Marine fish (times/month, mean ± SD)	7.34 ± 9.47	7.26 ± 8.67	7.51 ± 9.96	7.28 ± 9.97	0.975^b^
Marine shrimp/crabs (times/month, mean ± SD)	2.34 ± 5.49	2.47 ± 5.32	2.42 ± 5.95	2.11 ± 5.28	0.847^b^
Marine shellfish (times/month, mean ± SD)	1.30 ± 3.99	1.24 ± 3.50	1.56 ± 5.22	1.12 ± 3.12	0.682^b^
Freshwater fish (times/month, mean ± SD)	6.15 ± 9.16	5.40 ± 9.23	6.72 ± 9.37	6.49 ± 8.87	0.454^b^
Freshwater shrimp/crabs (times/month, mean ± SD)	1.85 ± 5.55	2.03 ± 7.00	1.78 ± 4.44	1.72 ± 4.54	0.889^b^
Freshwater shellfish (times/month, mean ± SD)	1.06 ± 4.54	1.38 ± 6.25	0.91 ± 2.87	0.82 ± 3.29	0.563^b^
Cephalopods (times/month, mean ± SD)	0.89 ± 3.70	0.98 ± 3.86	0.76 ± 3.53	0.91 ± 3.71	0.890^b^
Eggs (times/month, mean ± SD)	21.23 ± 11.90	22.07 ± 10.79	22.16 ± 12.52	19.38 ± 12.42	0.124^b^
Dairy products (times/month, mean ± SD)	11.60 ± 13.57	12.47 ± 14.27	11.16 ± 13.70	11.00 ± 12.64	0.622^b^
Bean products (times/month, mean ± SD)	8.70 ± 11.16	8.23 ± 11.94	8.96 ± 10.57	9.01 ± 10.81	0.815^b^
Pickled vegetables (times/month, mean ± SD)	5.23 ± 12.06	5.60 ± 12.74	4.73 ± 11.06	5.27 ± 12.24	0.846^b^
Bacon products (times/month, mean ± SD)	1.28 ± 5.64	1.13 ± 4.14	2.34 ± 8.97	0.45 ± 1.42	0.019^b^

NC, normal cognition; CD, cognitive decline; MCI, mild cognitive impairment; SD, standard deviation.

^a^Chi-square test was used for the comparisons of categorical variables.

^b^One-Way ANOVA was used for the comparisons of continuous variables.

### Detection frequency and concentration of NDMA in urine

*N*-nitrosodimethylamine was detected in 55.4% (212/383) of urine samples, including 63.2% (91/144) in the NC group, 53.4% (62/116) in the CD group and 48.0% (59/123) in the MCI group (*P* = 0.039) (Table 4). The concentration of urinary NDMA varied greatly, ranging from 1.20 × 10^–7^ to 157.39 μg/L, with a median concentration of 0.23 μg/L. The concentration distribution of urinary NDMA among NC, CD, and MCI groups was significantly different (*P* = 0.022). The NC group had the highest median concentration (0.32 μg/L), followed by the CD group (0.27 μg/L), and the MCI group (0 μg/L). Moreover, there was a significant trend of concentration difference (*P*_*trend*_ = 0.024).

**TABLE 4:** Detection frequency and concentration of urinary *N*-nitrosodimethylamine by cognitive function status.

Variables	Total (*n* = 383)	Status of cognitive function			*P*-values
		**NC (*n* = 144)**	**CD (*n* = 116)**	**MCI (*n* = 123)**	
NDMA detection (*n*, %)					0.039^a^
No	171 (44.6)	53 (36.8)	54 (46.6)	64 (52.0)	
Yes	212 (55.4)	91 (63.2)	62 (53.4)	59 (48.0)	
Urine NDMA (μg/L, median, IQR)^c^	0.23 (0, 0.62)	0.32 (0, 0.74)	0.27 (0, 0.56)	0 (0, 0.57)	0.022^b^

NC, normal cognition; CD, cognitive decline; MCI, mild cognitive impairment; NDMA, N-nitrosodimethylamine; IQR, interquartile range.

^a^Chi-square test was used for the comparisons of categorical variables.

^b^Brown-Mood Median test was used for the comparisons of median variables.

^c^Jonckheere–Terpstra test was used to compare trends of multiple groups. *P* = 0.024 for concentration trend test.

### Association of urinary NDMA and cognitive function

Kendall’s Tau-b correlation analysis was used to assess the relationship between urine NDMA and cognitive function in the elderly community of Shenzhen. The results showed that urinary NDMA concentration had a strong negative correlation with cognitive impairment (*Kendall’s Tau-b* = −0.89, *P* = 0.024).

To investigate factors influencing urinary NDMA, variables with differences were incorporated into the disordered multiple classification Logistic regression analysis model for further analysis (Table 5). The results showed that the model with the addition of independent variables fit better than the model with only constant terms, and the improvement of the model was statistically significant (*P* < 0.001). Compared with the NC group, educational level, passive smoking and fresh vegetables were statistically significant in the CD group (all *P* < 0.05). Compared with NC group, educational level, length of residence and fresh vegetables were statistically significant in the MCI group (all *P* < 0.01). However, no differences were found in NDMA concentration or detection frequency.

**TABLE 5:** Parameter evaluation results by the disordered multiple classification logistic regression analysis model.

Group^a^	Variables^b^	*B*	SE	*Wald*	*df*	*P*	Exp(B)	Lower 95% CI of exp(B)	Upper 95% CI of exp(B)
**CD**									
	Intercept	−1.673	0.648	6.670	1	0.010			
	Gender 1	−0.091	0.286	0.101	1	0.750	0.913	0.521	1.599
	Gender 2	0^c^			0				
	Educational level 1	1.212	0.346	12.247	1	0.000	3.360	1.704	6.624
	Educational level 2	0.243	0.337	0.519	1	0.471	1.275	0.658	2.469
	Educational level 3	0^c^			0				
	Length of residence 1	0.418	0.398	1.106	1	0.293	1.520	0.697	3.314
	Length of residence 2	0.193	0.373	0.268	1	0.605	1.213	0.584	2.519
	Length of residence 3	0.351	0.429	0.667	1	0.414	1.420	0.612	3.293
	Length of residence 4	0^c^			0				
	Household registration 1	−0.009	0.397	0.001	1	0.981	0.991	0.455	2.158
	Household registration 2	0^c^			0				
	Passive smoker 1	−0.856	0.383	4.997	1	0.025	0.425	0.200	0.900
	Passive smoker 2	0^c^			0				
	Rice	0.010	0.007	1.701	1	0.192	1.010	0.995	1.024
	Fresh vegetables	0.019	0.008	5.417	1	0.020	1.019	1.003	1.036
	Bacon products	0.034	0.025	1.819	1	0.177	1.035	0.985	1.088
	NDMA detection 1	0.451	0.356	1.606	1	0.205	1.570	0.782	3.152
	NDMA detection 2	0^c^			0				
	NDMA concentration	0.199	0.118	2.859	1	0.091	1.220	0.969	1.536
**MCI**									
	Intercept	−1.440	0.665	4.690	1	0.030			
	Gender 1	−0.533	0.287	3.457	1	0.063	0.587	0.335	1.029
	Gender 2	0^c^			0				
	Educational level 1	0.606	0.372	2.654	1	0.103	1.833	0.884	3.798
	Educational level 2	0.958	0.318	9.066	1	0.003	2.605	1.397	4.859
	Educational level 3	0^c^			0				
	Length of residence 1	0.425	0.410	1.071	1	0.301	1.529	0.684	3.417
	Length of residence 2	0.121	0.394	0.093	1	0.760	1.128	0.521	2.444
	Length of residence 3	1.306	0.409	10.191	1	0.001	3.691	1.656	8.230
	Length of residence 4	0^c^			0				
	Household registration 1	0.655	0.401	2.667	1	0.102	1.926	0.877	4.227
	Household registration 2	0^c^			0				
	Passive smoker 1	−0.551	0.404	1.864	1	0.172	0.576	0.261	1.272
	Passive smoker 2	0^c^			0				
	Rice	−0.009	0.007	1.408	1	0.235	0.992	0.978	1.006
	Fresh vegetables	0.026	0.008	10.609	1	0.001	1.027	1.011	1.043
	Bacon products	−0.104	0.060	2.955	1	0.086	0.901	0.801	1.015
	NDMA detection 1	0.309	0.357	0.750	1	0.386	1.363	0.677	2.745
	NDMA detection 2	0^c^			0				
	NDMA concentration	0.181	0.118	2.350	1	0.125	1.198	0.951	1.510

NC, normal cognition; CD, cognitive decline; MCI, mild cognitive impairment; CI, confidence interval.

^a^The reference category is NC group.

^b^Please refer to Supplementary Table 1 for variables assignment.

^c^The reference variable.

### EDI of NDMA and health risk assessment

The median EDI of NDMA were calculated to be 6.63 ng/kg-bw/day for the elderly Chinese subjects. The 5th percentile EDI and 95th percentile EDI of NDMA was 3.56 × 10^–6^ ng/kg-bw/day and 86.24 ng/kg-bw/day, respectively. The median HQ of cognitive impairment from NDMA exposure was calculated to be 33.15. These results indicated that NDMA exposure may have a certain impact on cognitive function in the Chinese elderly population.

## Discussion

### Overview

Whereas nitrosamines and their analogs are known carcinogens, the present animal and human studies suggest for the first time that cognitive function may be altered by NDMA exposure. Mice sub-chronically treated with NDMA displayed largely dose-dependent lower scores in a behavioral test of learning and memory, albeit in the absence of detectable structural changes in the brain. Human studies revealed an association with cognitive dysfunction in elderly subjects consuming nitrosamine-contaminated food items but, surprisingly, the median urinary concentration of NDMA was inversely related to the degree of cognitive deficit. The explanation for this result may lay in the origin and metabolic fate of endogenous NDMA in human subjects, which includes the formation of formaldehyde (Yoo et al., 1988).

### Exogenous and endogenous sources of NDMA

The oral route, including consumption of contaminated food and water, is the primary human exposure pathway for NDMA (U.S. Environmental Protection Agency, 2014). The median EDI of NDMA obtained in the present study (6.63 ng/kg-bw/day) was lower than 114 ng day(−1) dietary intakes in the Spanish cohort (Jakszyn et al., 2006) but higher than 1.08 × 10^–6^ mg/(kg*d) chronic daily intake from food sources in China (Sang et al., 2019). We detected NDMA in ∼10% of purchased food items, approximately one third of which (mainly seafood items) exceeded the Chinese National Standard Limit Value. Approximately one half of each of the three groups of elderly human subjects had detectable urinary NDMA, the median concentration of which was 0.234 μg/L. This compares with NDMA levels measured in urine samples from residents of Anhui province in China (0.27 nmol/g creatinine (Guo et al., 2013) and Atlanta, Georgia in the United States (geometric means of 49.22 pg/mL for non-smokers and 62.58 pg/mL for smokers (Seyler et al., 2013). Reported cigarette smoking was comparable across our three test groups, although mean levels of exposure to passive cigarette smoke was significantly higher among the CD group and the MCI group vs. the NC group.

While NDMA exposure of our test participants likely occurred variably by inhalation of cigarette smoke and intake of contaminated food and drinking water (Chen et al., 2019), it is important to note that exogenous sources of NDMA contribute only a component of total endogenous NDMA. Sang et al. (2019) found that the contribution rates of drinking water and food sources accounted for 0.08 and 0.69% of endogenous NDMA in Chinese subjects. Endogenous NDMA also arises from the formation of NDMA *in vivo* from the consumption of meat and bacon cured with nitrite, nitrate-rich vegetables and seafood (Laitinen et al., 1993; Dich et al., 1996; Zeilmaker et al., 2010). Post-mortem study of human subjects showed that endogenous NDMA is found in all tissues, including the brain (Cooper et al., 1987). Data from population studies (Vermeer et al., 1998; Seyler et al., 2013; Hodgson et al., 2016) indicate that NDMA is commonly detected in urine, and the levels of NDMA are raised by consumption of food rich in nitrate and amines (van Maanen et al., 1998).

### Metabolism of NDMA

Human and rodent metabolism of NDMA uses the cytochrome P450 system in liver microsomes (Yoo et al., 1988; Fournier, 1990). There are two separate routes of oxidative metabolism: denitrosation that produces nitrite and demethylation that produces formaldehyde. Higher endogenous levels of NDMA increase the ratio of demethylation:denitrosation. While formaldehyde is a physiological molecule that participates in the one-carbon cycle, the endogenous level of formaldehyde must be strictly regulated. Chinese investigators have demonstrated that formaldehyde is associated with deficits in human cognition, such that there is a correlation between urine formaldehyde and cognitive abilities throughout the AD continuum (i.e., formaldehyde levels in AD > MCI > early cognitive decline). Urine formaldehyde levels also correlated with gender, plasma Aβ_42_ and p-Tau181/T-tau (markers of AD) (Wang et al., 2022).

NDMA demethylation, followed by non-enzymatic cleavage of the hydroxylated methyl group produces formaldehyde and methyldiazohydroxide, which then leads to the formation of a methonium ion that methylates nucleophilic sites of cellular macromolecules such as proteins, RNA and DNA. DNA adducts so formed include *O*^6^-methylguanine and *N*^7^-methylguanine, the same DNA lesions generated by the developmental neurotoxin and carcinogen MAM which, like NDMA, is also metabolized to formaldehyde and methonium (carbonium) ion (Feinberg and Zedeck, 1980). MAM, which served as a positive control for NDMA in the present animal study, is etiologically associated with ALS/PDC, a prototypical neurodegenerative disease (Spencer et al., 2020).

P-450 enzymes efficiently catalyze the demethylation of NDMA (Tu and Yang, 1985). P-450-mediated metabolism of NDMA is complex, but P450 2E1 (encoded in humans by the *CYP2E1* gene) has the lowest Km (Sulc et al., 2010), which corresponds to the highest affinity for the substrate (i.e., NDMA). One testable possibility, therefore, is that MCI subjects (low urinary NDMA) have a high endogenous expression of P450 2E1 that produces high levels of circulating formaldehyde resulting in cognitive decline. If correct, normal (NC) subjects would be expected to have relatively low P450 enzyme activities such that the reduced rate of NDMA demethylation allowed the compound to be excreted in relatively high concentration in urine. While P450 2E1 is an ethanol-inducible enzyme (Robin et al., 2005), we found no difference in the reported use of ethanol among the three study groups. Nevertheless, if genomic expression of *CYP2E1* varied among these subjects, this might explain why urine NDMA levels in NC > ND > MCI. Genetic variation in *CYP2E1*, which has been documented in the Chinese population (Huang et al., 2012), is an established cause of significant inter-individual differences in drug response and the risk of neurodegenerative disease (Sheng et al., 2021). Future studies examining NDMA metabolism should also determine the enzyme activity of alcohol dehydrogenase 5 (ADH5), which converts formaldehyde to formate, a less reactive molecule used in nucleotide biosynthesis (Reingruber and Pontel, 2018).

### Study limitations

While this study found that urinary NDMA concentration had a strong negative correlation with cognitive impairment, Logistic regression analysis found that NDMA concentration or detection frequency did not significantly contribute to the model maybe because of sample size or confounding factors, this phenomenon and its underlying cause requires further study. Additionally, we showed a direct relationship between exogenous NDMA administration and cognitive changes in mice. But this result was based on a single test system, no significant pathological changes were found in vulnerable areas of the brain, and immunocytochemical indicators for abnormal neuroprotein deposition (e.g., Aβ and Tau) were not addressed.

## Conclusion

This study is the first to analyze urinary NDMA exposure levels in the elderly population of a large city in southern China. There was a strong negative correlation between urinary NDMA levels and cognitive impairment. Animal experiments showed that subchronic NDMA treatment impairs learning and memory function. Together, these should be treated as preliminary findings that require confirmation and further analysis.

## Data availability statement

The raw data supporting the conclusions of this article will be made available by the authors, without undue reservation.

## Ethics statement

The studies involving human participants were reviewed and approved by the Ethics Committee of Shenzhen Center for Disease Control and Prevention. The patients/participants provided their written informed consent to participate in this study. The animal study was reviewed and approved by the Ethics Committee of Shenzhen Center for Disease Control and Prevention.

## Author contributions

JL and PS contributed to the conception of the study and designed the experiments. WL contributed significantly to collect and analysis the data and drafted the manuscript. ZY did the routine analyses of food and urine samples. JH, GH, and XC did the survey and the animal experiments. ZW, YL, and PS performed the analysis with constructive discussions and revised the manuscript. All authors read and approved the final manuscript.
