# Supplementary material for: Association of N-nitrosodimethylamine exposure with cognitive impairment based on the clues of mice and humans
Source: Front Aging Neurosci. 2023 Jun 27;15:1137164. doi: 10.3389/fnagi.2023.1137164 (PMC10333700; doi:10.3389/fnagi.2023.1137164)
Supplement: Supplementary file 1 [file Table_1.docx]

Supplementary Table 1 Variables assignment in the disordered multiple classification Logistic regression analysis model.

| **Variables** | **Assignment** |
| --- | --- |
| Gender | 1=Male, 2=Female |
| Educational level | 1=Primary school or illiteracy, 2=Middle school, 3=High school or higher |
| Length of residence | 1=1-9 year, 2=10-19 year, 3=20-29 year, 4=30-78 year |
| Household registration | 1=No, 2=Yes |
| Passive smoker | 1=No, 2=Yes |
| Rice | Raw data |
| Fresh vegetables | Raw data |
| Bacon products | Raw data |
| NDMA detected | 1=No, 2=Yes |
| NDMA concentration | Raw data |
| Cognitive impairment | 1=NC, 2=CD, 3=MCI |
